# Fatty Acid Allosteric Regulation of C-H Activation in Plant and Animal Lipoxygenases

**DOI:** 10.3390/molecules25153374

**Published:** 2020-07-24

**Authors:** Adam R. Offenbacher, Theodore R. Holman

**Affiliations:** 1 Department of Chemistry, East Carolina University, Greenville, NC 27858, USA; 2 Department of Chemistry and Biochemistry, University of California Santa Cruz, Santa Cruz, CA 95064, USA; holman@ucsc.edu

**Keywords:** protein allostery, C-H activation, kinetic isotope effects, substrate selectivity, hydrogen tunneling

## Abstract

Lipoxygenases (LOXs) catalyze the (per) oxidation of fatty acids that serve as important mediators for cell signaling and inflammation. These reactions are initiated by a C-H activation step that is allosterically regulated in plant and animal enzymes. LOXs from higher eukaryotes are equipped with an N-terminal PLAT (Polycystin-1, Lipoxygenase, Alpha-Toxin) domain that has been implicated to bind to small molecule allosteric effectors, which in turn modulate substrate specificity and the rate-limiting steps of catalysis. Herein, the kinetic and structural evidence that describes the allosteric regulation of plant and animal lipoxygenase chemistry by fatty acids and their derivatives are summarized.

## 1. Introduction

Lipoxygenases (LOXs) are widely represented in plants, animals, fungi, and select bacteria [1]. These enzymes catalyze the (per) oxidation of fatty acids, forming a diverse array of products in which the physiological roles exhibit considerable variability depending upon the organism.

**Figure 1:**
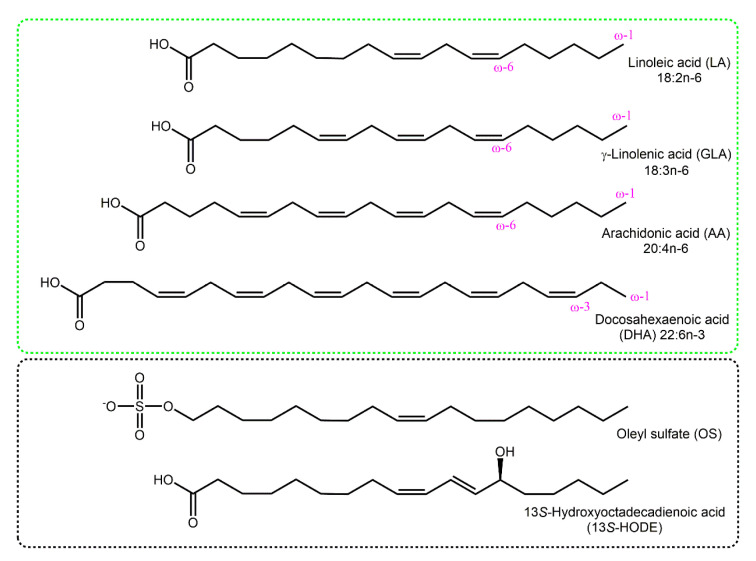
Structures of select substrates (green box) of lipoxygenase (LOX) and two relevant allosteric effectors (black box) as discussed in the text.

In plants, LOX is present in many species and is involved in a number of critical functions such as seed germination, growth, development, and pathogenic defense [2]. One such species, soybean, has six isoforms, with soybean LOX-1 (SLO-1) emerging as a robust model system due to its ease of production and stable enzymatic properties. The fatty acid composition of plants is comprised of simple poly-unsaturated fatty acids (PUFAs) such as linoleic acid (LA, C18:2) and gamma-linolenic acid (GLA, C18:3), and thus LA is the most commonly used fatty acid substrate (Figure 1). SLO-1 oxygenates LA on C13 to produce 13*S*-hydroperoxyoctadecadienoic acid (13S-HpOD) in both a regiospecific and stereospecific manner [3,4]. Parenthetically, SLO-1 is often referred to as a 15-LOX because it oxygenates the ω-6 carbon, which is C13 on LA, but C15 on arachidonic acid (AA, C20:4).

Human LOXs also have important biological roles, with the six isoforms being involved in normal homeostasis, inflammation, and the pro-resolution response [5,6]. One biological aspect of human LOXs that make them especially challenging to study is the plethora of PUFA substrates available in the body and the dramatically different biological functions their products perform. For example, AA, an ω-6 PUFA, can be oxygenated to hydroperoxyeicosatetraenoic acids (HpETEs), which can either bind to receptors directly or be converted to pro-inflammatory leukotrienes or pro-resolution lipoxins (Figure 1) [7]. Docosahexaenoic acid (DHA, C22:6), an ω-3 PUFA, is another substrate to LOXs, and yet its hydroperoxide products have distinct biological roles than those of AA (Figure 1) [8,9]. Amazingly, the human body can contain over 30 distinct PUFAs, depending on the individual’s diet, which can react with the six human LOX isozymes. Therefore, it is easy to hypothesize that from a strictly substrate availability perspective, the regulation of the biosynthetic web of human LOXs would be quite complex. To add to this complexity, it is possible that LOXs are also allosterically regulated by their own LOX products in the cell, making not only the understanding of their biological roles more complicated, but also the consequences of their inhibition as well.

The development of therapeutics that target human LOXs is a key challenge for the understanding of LOXs role in human disease; however, there is only one FDA-approved anti-inflammatory LOX drug, Zileuton [10]. Zileuton targets human 5-lipoxygenase (h5-LOX) and is a hydroxyamide whose inhibitory mechanism of action is through both a metal chelation and reduction. The reason for this dearth of LOX therapeutics in general is multifaceted, but two limitations in the advancement of LOX-targeting anti-inflammatory inhibitors has been the characterization of the specific role of each LOX isozyme and the difficulty in identifying/developing isoform-selective inhibitors. However, with recent successes, this issue is slowly being overcome [11,12,13,14,15,16,17,18,19].

LOX inhibitors can be classified into competitive and reductive inhibitors. The reductive inhibitors, while very potent, are less desirable due to their potential chemical modification in the changing redox environment of the cell. For the orthosteric inhibitors, they are typically not purely competitive, but rather display mixed inhibition, which raised the possibility of an allosteric site. This hypothesis was confirmed, when both plant and animal LOXs were shown to exhibit allosteric control, either through activation, regulation of substrate selectivity and/or the rate-limiting steps of catalysis. These results indicate that allosteric regulators of human LOXs could be developed as “partial” inhibitors and not “on/off” competitive inhibitors, which could be highly beneficial since LOXs are involved in both pro-inflammatory and pro-resolution pathways [6].

This mini review will focus on the allosteric regulation of plant and animal LOXs, particularly by fatty acids and their derivatives. Experimental evidence from select model LOX systems will be summarized, with SLO-1 representing plant LOXs and the human reticulocyte 15-LOX-1 (h15-LOX-1), human epithelial 15-LOX-2 (h15-LOX-2), and human platelet 12-LOX (h12-LOX) representing the animal LOXs.

## 2. Structures of Lipoxygenases

### 2.1. Catalytic Domain

**Figure 2:**
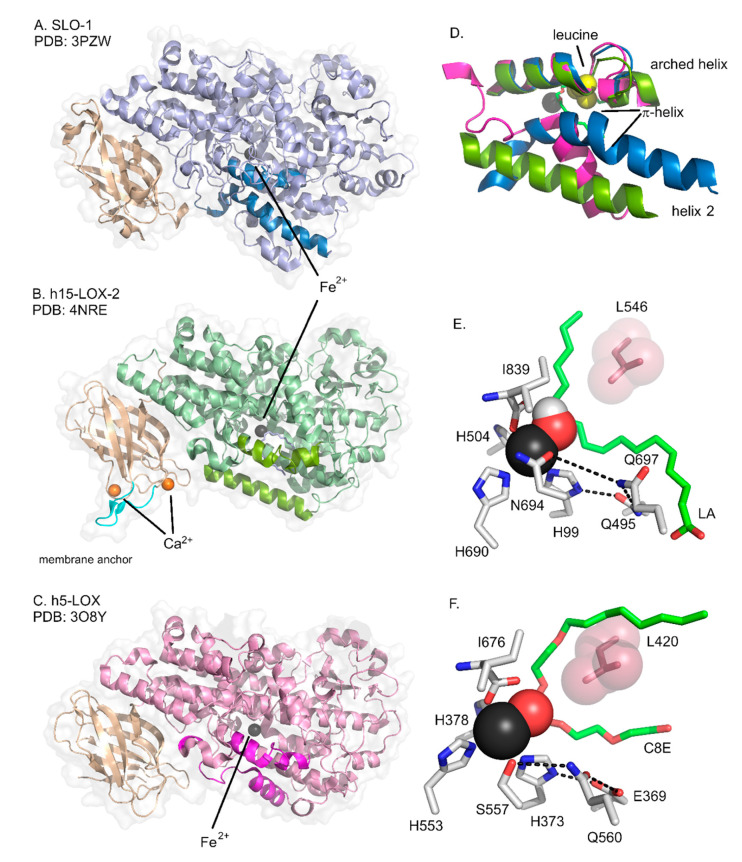
Structural comparison of SLO-1 (**A**), h15-LOX-2 (**B**), and h5-LOX (**C**). The catalytic domains are colored blue, green, and pink, respectively; the catalytic metal is represented as a black sphere. For both plants and animals, the N-terminal PLAT (Polycystin-1, Lipoxygenase, Alpha-Toxin) domain is colored in wheat. The PDB identification numbers of the structure are listed for reference. The arched helix and helix 2, discussed in text, are colored dark blue (**A**), split pea (**B**), and magenta (**C**), and reproduced in (**D**). In panel (**D**), the image was rotated 30° relative to (**A**–**C**). The position of the invariant leucine is represented in yellow spheres. For reference, the positioning of the substrate mimic solved in h15-LOX-2 is represented as green sticks. Panels (**E**,**F**) show the coordination environment surrounding the iron water (black and red sphere, respectively) cofactor for (**E**) SLO-1 and (**F**) h15-LOX-2 active site structures. The hydrogen-bonded networks are represented as dashed lines. The SLO-1 substrate, LA (green), was modeled into the active site using electron nuclear double resonance (ENDOR) distance restraints [21]. The h15-LOX-2 structure was solved with a substrate mimic, octyltetraethylene glycol ether (C8E). There are no structures of full length h12-LOX or h15-LOX-1.

The predominantly alpha helical catalytic domain (Figure 2A–C) is structurally maintained in all lipoxygenases and is approximately 500–700 amino acids in length [9]. Plant LOXs are typically larger in size and decorated with additional surface loops. Despite the structural similarity, LOXs have relatively poor sequence homology. Pairwise identity among plant sequences is 43–86%, and identity among paired mammalian sequences is 39–93% [20]. The clustering of alpha helices in the catalytic domain is nearly the same with the notable exception of helix 2 (Figure 2A–D), which resides parallel to an arched helix lining the substrate cavity (Figure 2D). Buried within the core of the catalytic domain is a hydrophobic substrate binding channel that is U-shaped in animal LOXs to accommodate AA, though less bent in plant enzymes, possibly to accommodate the more saturated LA.

The original crystal structure of SLO-1 (PDB: 2SBL; 2.6 Å) was the first LOX structure solved [22,23]. This information helped to shed light into the global architecture of the protein and coordination environment surrounding the catalytic cofactor (Figure 2E). A few years later, a high-resolution (1.4 Å) structural model of SLO-1 was reported [24], with the putative substrate portal proposed to be gated by residues T259 and K260 on helix 2 and L541 on the arched helix. Since then, dozens of SLO-1 crystal structures have been solved (see references [21,25,26,27,28], for example), though all are devoid of substrate (analogs) or allosteric effectors. Another isoform, SLO-3, was resolved with the product 13*S*-hydroperoxyoctadecadienoic acid (13*S*-HpOD) filling the substrate pocket [29]. However, significant alterations to the ligand environment surrounding the metal center were observed in this structure, including a direct coordination of the hydroperoxide product to the metal center. The head-to-tail orientation of the product and its relevance to productive substrate binding in SLO-1 remains contentious [21,30,31,32,33].

Pulsed electron paramagnetic (dipolar) spectroscopy experiments performed using a combination of strategic protein conjugated nitroxide spin labels and a spin-labeled substrate mimic mapped the entrance of the SLO-1 substrate portal [34,35]. Triangulation of 15 spin couplings determined that the head group of the substrate mimic resides near residues E236, K260, and Q264 along helix 2 and residue Q544 along the arched helix in SLO-1 [34], which is nearly the same binding site as that predicted from the substrate-free structure [24]. Residues 260 and 264 are located near or within the six amino acid segment that comprises a π-helix (residues 261–267), whose structure and dynamics are sensitive to substrate binding [35,36]. π-helices are rare in proteins and are comprised of an additional amino acid residue inserted into a canonical α-helix. As such, these structural features destabilize α-helices, which in turn impart different (or enhanced) functionality through the evolution of protein structure [20]. The rarity of π-helices manifests in the fact that the π-helix, embedded in helix 2, is only found in plant LOXs.

The structure of the human 5-LOX enzyme without substrate (analog) exhibits an occluded active site, shielded by bulky aromatic amino acids, referred to as a “cork”, on helix 2 (Figure 2C,D, magenta) [37]. The location of this “cork” in h5-LOX is near the position of the π-helix in SLO-1 (Figure 2D). It has been suggested that, in order for a substrate to bind, helix 2 of h5-LOX must undergo a conformational change [37,38]. As observed by mutagenesis, the bulk of tyrosine at position 181 may provide the “cork” to limit substrate entrance, as substitution of Y181 for alanine enhances catalytic proficiency threefold [39]. Furthermore, structures of h15-LOX-2 and coral 8*R*-LOX were determined with substrate (mimics) in their active sites [40,41]. Based on these structures (cf. Figure 2D), the substrate entrance portal is consistent with that described previously for SLO-1. The aggregate structural data of LOX isozymes support that the location of the substrate entrance portal is near helix 2 and likely in the same location across plants and animal enzymes, despite their low sequence identity.

The conformation (and dynamics) of helix 2 may play an important, though isoform-dependent, role in substrate acquisition [41]. For h5-LOX, the structure of the apo (substrate-free) enzyme (Figure 2C) has an occluded active site. The helix 2 (residues 169–201) of h5-LOX in this model features a short three turn helix flanked by loops, whereas the helix 2 elements of h15-LOX-2 and SLO-1 are comprised of contiguous six to seven turn helices (Figure 2D). A recent structure of an inhibitor h5-LOX complex [38] revealed uninterpretable electron density for residues 173–214, including a significant portion of helix 2. Together, the results were interpreted in the context of an inhibitor-induced (or ligand-induced) change in helix 2 conformation. Conversely, there is no obvious structural differences in helix 2 conformation between substrate-free SLO-1 (PDB: 3PZW) and product-bound SLO-3 (PDB: 1IK3). Adjacent to the substrate binding channel resides the catalytic iron metallocofactor. The coordination environment surrounding the iron center is almost completely conserved with three histidine residues, the carboxylate from the C-terminus, and the catalytic water/hydroxide molecule (Figure 2E). The sixth axial ligand varies among LOX isozymes, being a weak asparagine ligand in SLO-1 and h12-LOX, a weak serine for h15-LOX-2, and a strong histidine for h15-LOX-1 [40,42]. The sixth Asn/His/Ser axial ligand also participates in a second-sphere hydrogen-bonded network (Figure 2E,F), which influences the coordination of this ligand and, by extension, the reactivity of the metallocentre, as seen by mutagenesis [25].

As discussed above, parallel to helix 2 is an arched helix that covers the substrate binding channel (Figure 2A–D). Note the slight variation in substrate positioning between LA in SLO-1 and a co-crystalized substrate mimic in h15-LOX-2. This arched helix holds an invariant leucine that is responsible for proper substrate positioning (Figure 2E,F, firebrick red spheres). Mutation of L546 in SLO-1 or L407 in h12-LOX to the volume-reducing side chain, alanine, resulted in a ca. 100-fold decrease in catalytic proficiency in either case [43,44]. These mutations do not greatly change the redox potential of the metal center, but perturb the proper positioning of substrate binding with respect to the catalytic center [28,44].

### 2.2. PLAT Domain

Plant and animal LOXs exclusively have an additional domain at their N-terminus, labeled a PLAT (Polycystin-1, Lipoxygenase, Alpha-Toxin) domain, which is arranged as a C2-like β-barrel. C2 domains are only found in higher eukaryotes and have demonstrated both Ca^2+^-dependent and Ca^2+^-independent membrane binding capabilities [45,46]. By extension, the N-terminal PLAT domain in plant and animal LOXs has been suggested to be involved in membrane association. Though the structures of h5-LOX do not have Ca^2+^ bound (Figure 2C) [37,38], there is strong evidence to support calcium-dependent membrane binding. Cellular experiments, in which the N-terminal β-barrel of human 5-LOX was expressed independently of the catalytic domain, established the localization of the PLAT domain to the lipid membrane [47]. Removal of the PLAT domain or introduction of specific mutations is often accompanied by structural instability of the catalytic domain, altered catalytic rates, and/or altered substrate specificity [48,49,50,51]. 

In select mammalian LOXs, calcium binding at designated loops within the PLAT domain is associated with activation of enzymatic activity [47,52,53,54]. The structure of h15-LOX-2 was seen to contain two Ca^2+^ ions bound in the PLAT domain (Figure 2B, orange spheres) [40]. The Ca^2+^ ions in the h15-LOX-2 structure appear to stabilize a membrane insertion loop (Figure 2B, cyan) that is anticipated to anchor the enzyme to the lipid bilayer [55]. Comparative X-ray structures of mammalian LOXs in the presence and absence of Ca^2+^ show changes to the loop structures in the PLAT domain. Hydrogen/deuterium exchange experiments of h15-LOX-2 supported subtle conformational changes to this membrane insertion loop when Ca^2+^ was added to the samples [56]. Because of the significant variability in the primary sequences and loop structures of the PLAT domain, calcium regulation does not appear ubiquitous. For example, SLO-1 reactivity is not dependent upon calcium [57], though high Ca^2+^ concentrations have been shown to elicit structural fluctuations in the catalytic domain [58].

## 3. The Lipoxygenase Reaction

### 3.1. General Mechanism

**Scheme 1:**
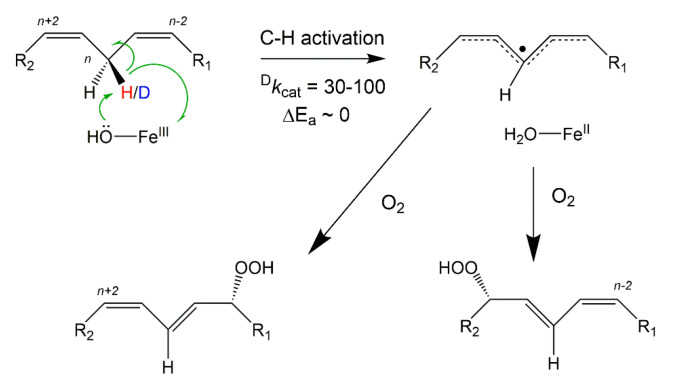
General mechanism of fatty acid oxidation by iron LOXs. The reaction is initiated by a hydrogen atom abstraction at carbon *n* and molecular oxygen insertion occurs antarafacially either at the *n* + 2 or *n* − 2 carbon. In the case of SLO-1, the pro-S hydrogen (red) is abstracted from carbon-11 (*n*) of linoleic acid (LA), leading to oxygen insertion at (predominantly) the *n* + 2 carbon, to form 13*S*-HpOD. For h15-LOX-2, C-H cleavage occurs at carbon-13 of AA with oxygen insertion at *n* + 2 carbon to form 15*S*-HpETE.

The initial and often rate-limiting step for the oxidation of fatty acids by lipoxygenases is a C-H activation (C-H bond cleavage) step (Scheme 1) [59]. This process occurs by proton-coupled electron transfer (PCET) in which the proton is transferred to the metal-bound hydroxide and the electron is accepted by the metal center, converting it from the +3- to the +2-oxidation state [30,60]. This C-H cleavage step results in a delocalized radical species that is subsequently quenched by molecular oxygen. The ensuing hydroperoxide product is generated through a reverse PCET step followed by release of the product and restoration of the catalytic metallocofactor. 

For the majority of LOXs, the metal source is a mononuclear, non-heme iron. Substitution of this metal for other redox-active metals (e.g., Ni^2+^, Mn^2+^, Co^2+^) renders the enzyme virtually inactive [61,62]. The oxidation state of the metal in the LOX isolated from the native source or recombinantly is in the +2 state, but this oxidation state is inactive. The enzymes require activation through treatment of a hydroperoxide enzyme product such as 13*S*-HpOD [63,64].

Under atmospheric oxygen concentrations, oxygen insertion into the delocalized radical is often regio- and stereo-selective [1,65,66]. For iron LOXs, abstraction of the hydrogen atom at carbon *n* of a particular PUFA results in the addition of oxygen at either carbon *n* − 2 or carbon *n* + 2 (Scheme 1). Toward this end, molecular oxygen insertion occurs antarafacially (i.e., on the opposite face of hydrogen abstraction). Furthermore, many LOXs earn their nomenclature from their prominent product. For example, the human LOXs (h5-LOX, h12-LOX, and h15-LOX-1/2) are so named because of the position of the oxygen insertion into arachidonic acid (AA). The carbon selectivity is achieved through proper positioning (and orientation) of the substrate within the channel, the utility of defined oxygen channels, and Coffa–Brash molecular determinants [41,67,68].

### 3.2. C-H Activation by Tunneling

**Figure 3:**
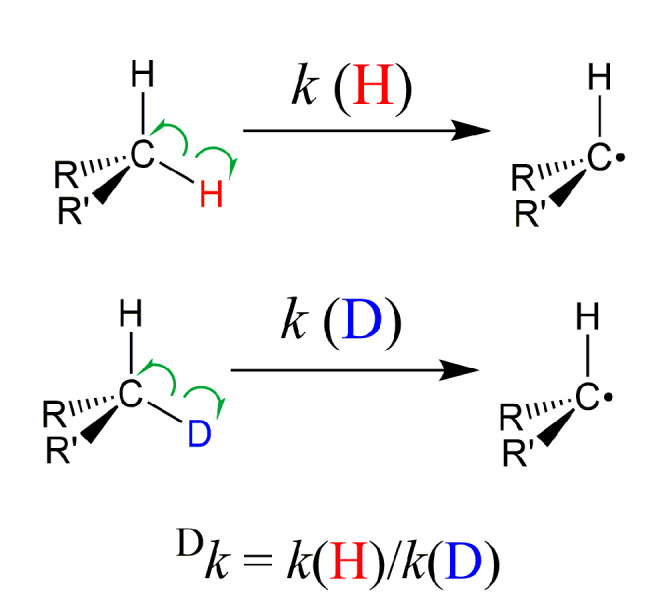
Homolytic C-H/C-D bond cleavage (formal H• transfer) as carried out by lipoxygenases and the determination of kinetic isotope effects (^D^*k*). Note that C-H bonds can also be cleaved through heterolytic processes, resulting in proton (H^+^) and/or hydride (H^−^) transfer. However, these enzymatic processes are often associated with modest kinetic isotope effects [78]. Isotope effects can be assessed for the first-order (^D^*k*_cat_) or second-order (^D^*k*_cat_/K_M_) rate constants.

C-H activation (C-H bond cleavage, Figure 3) is a fundamental chemical reaction prevalent in many biological processes. Enzyme-catalyzed C-H activation can be mediated by a redox-active metallocofactor, such as iron, or by an organic centered radical formed through the transient oxidation of natural amino acids (e.g., tyrosine or cysteine), or a 5′-deoxyadenosyl radical derived from the homolytic cleavage of *S*-adenosyl methionine (SAM) [69,70]. The chemical diversity achievable through enzymatic C-H activation is considerable [71,72,73,74,75,76,77].

There is an accumulation of experimental data that supports non-classical (tunneling) behavior for these chemical reactions [78]. Regardless of species, the primary deuterium kinetic isotope effects on the first-order rate constant, ^D^*k*_cat_ = *k*_cat_(H)/*k*_cat_(D) (Figure 3), associated with LOX chemistry are in the range of 24–100 for the native enzymes [79,80,81,82,83]. These values are far in excess of the semi-classical limit of 7 and are suggestive of tunneling behavior. In contrast to the cleavage of O-H and N-H bonds, C-H bonds are not exchangeable with deuterated solvents. Through exploiting chemical syntheses, site-selective isotope substitution methods can be made to a single C-H bond to study bond-selective kinetic isotope effects (Figure 3).

**Figure 4:**
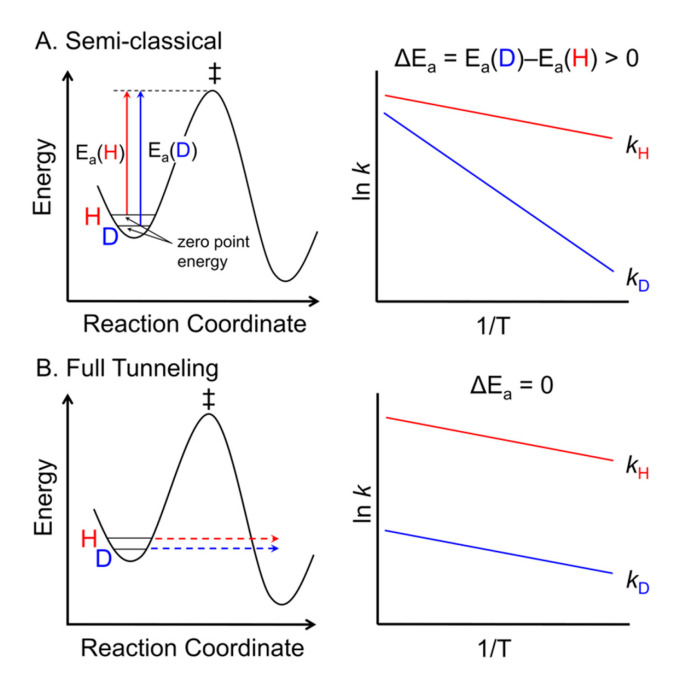
Reaction progress diagrams (left) and Arrhenius plots (right) for the cleavage of C-H/C-D bonds. Classical transition states are indicated by ‘‡’. (**A**) Representation of semi-classical origins of the kinetic isotope effect (KIE). (**B**) Representation of a full tunneling mechanism for hydrogen transfer through the barrier.

While large primary deuterium kinetic isotope effects, in excess of the semi-classical limit, implicate non-classical behavior, an important kinetic parameter to assign quantum tunneling mechanisms is the magnitude of the temperature dependence of the kinetic isotope effect (i.e., ΔE_a_ = E_a_(D) − E_a_(H)) [84]. From semi-classical reactions, ΔE_a_ values of greater than zero are expected due to the differences in the ground-state zero-point energies (Figure 4A). The observation of (near) zero values of ΔE_a_ cannot be rationalized by semi-classical models of catalysis but can be well fit to tunneling treatments for hydrogen transfer (Figure 4B). Numerous reports on enzyme systems on C-H activation, including lipoxygenases, have been associated with (near) zero ΔE_a_ values and are consistent with tunneling models [78]. For example, the SLO-1 reaction with LA exhibits a ^D^*k*_cat_ of approximately 80 that is nearly temperature-independent (ΔE_a_ = 0.9 ± 0.2 kcal/mol) [43]. For comparison, mutagenesis of single SLO active site aliphatic residues to tunneling-impairing, volume-reducing side chains have been associated with increases in the ΔE_a_ parameter as large as 5.3 kcal/mol [26,43]. The corresponding reaction of SLO-1 with the non-physiologically relevant AA is more complicated, but also results in a large ^D^*k*_cat_ of 97 ± 5 [85]. The ^D^*k*_cat_ values for h15-LOX-1/2 with LA and AA have been reported in the range of 40 to 50 and are temperature-independent [81].

Though large temperature-independent ^D^*k*_cat_ values have been determined for SLO-1 and h15-LOX-1/2, SLO-1 has served as the primary workhorse for nearly three decades with several critical control experiments conducted to conclude that the C-H activation step is rate-determining and irreversible, and thus permitting validation of the tunneling model. First, the K_M_ of molecular oxygen (second substrate) was found to be ≤30 μM, which lies well below the concentration of oxygen in ambient air (250–300 μM) [86,87]. Increasing the oxygen concentration to ~100% does not lead to altered SLO-derived products or kinetic parameters. These features ensure complete and rapid quenching of the delocalized fatty acid radical by oxygen, with no evidence for reversible C-H bond cleavage.

Early kinetic isotope effect (KIE) studies on lipoxygenase, acquired noncompetitively during steady-state turnover conditions for the dideuterated (11,11-D_2_) substrate, gave rise to the largest primary deuterium kinetic isotope effects at room temperature (~80). Competitive isotope effects measured using a discontinuous HPLC method resulted in comparable ^D^*k*_cat_ values to those determined via steady state [79]. Pre-steady state (single turnover) kinetics conducted under anaerobic conditions, which directly report on the transition of Fe^3+^ to Fe^2+^, also generated a large isotope that is linked to the C-H bond cleavage step [88]. At elevated temperatures, the magnitudes of the kinetic isotope effects for the first- and second-order rate constant converge [89], also supporting the hypothesis that the C-H bond cleavage step is rate-determining. The extraction of a secondary KIE from the synthesis of a monodeuterated 11*S*-D_1_ labeled LA exhibited a modest secondary isotope effect of ~1.2 for the non-transferred hydrogen [90].

**Figure 5:**
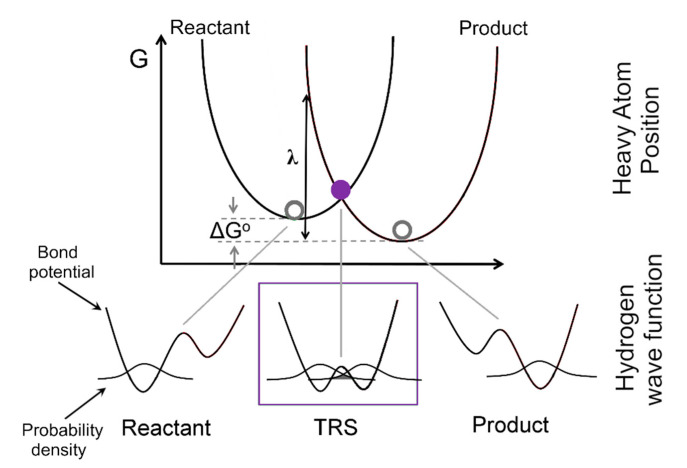
Graphical representation of the Marcus reaction coordinate involving the heavy atom positions and defined by λ and the driving force, ΔG^o^. At the tunneling ready state (TRS; filled purple dot), the positioning of the hydrogenic wave function has become degenerate across the donor and acceptor wells.

There are several important implications of hydrogen tunneling towards enzyme catalysis [59]. First is the breakdown of semi-classical transition state theory (over-the-barrier) view of catalysis. Because the process relies upon quantum (probability) wave function overlap, there is no classical transition state. Instead, the barrier is attributed to the stochastic search along the protein conformational landscape and the Marcus reorganization energy, λ, along the reaction coordinate (Figure 5).

Second, effective hydrogenic wave function overlap necessitates that the donor and acceptor must come into very close proximity. The unified view for C-H activation is that the internuclear distance between donor and acceptor approach 2.7–2.8 Å at the tunneling ready state (TRS) for efficient hydrogen tunneling [85,91,92,93]. In the case of SLO-1, there is no crystal structure with bound substrate. However, a high precision electron nuclear double resonance (ENDOR) spectroscopic study resolved the ground-state donor (carbon-11) and acceptor (metal-bound oxygen) distance as ca. 3.1 Å, consistent with the expectation of van der Waals contacts [21]. The reduction in this distance thus occurs transiently and may be facilitated by defined protein conformational networks involved in thermal activation [27,94]. The efficiency of active site compaction is influenced by proper steric bulk of hydrophobic residues at the active site [26,43,95,96].

### 3.3. Second-Order KIEs Indicate Substrate Binding as Partial Rate-Limiting at Lower Temperatures

**Figure 6:**
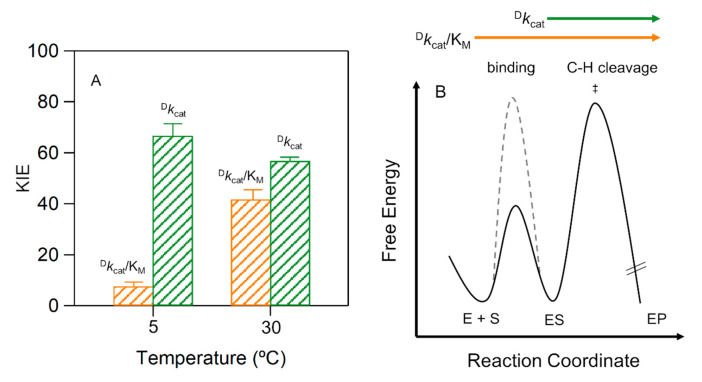
Kinetic isotope effects reported for the first-order (^D^*k*_cat_) and second-order (^D^*k*_cat_/K_M_) rate constants for the reaction of SLO with substrate LA, as a function of temperature (**A**) [99]. (**B**) Schematic representation of the reaction coordinate for SLO reaction with LA at 30 °C (solid line). The ‘‡’ represents the classical transition state. The decrease in ^D^*k*_cat_/K_M_, relative to ^D^*k*_cat_, observed at 5 °C is associated with an increase in the kinetic barrier for substrate binding (dashed line). Note that the ^D^*k*_cat_ values are smaller than 80, as originally reported [89]. These lower ^D^*k*_cat_ values were attributed to slight substrate contamination, and while the magnitudes of the ^D^*k*_cat_ are different, the trends are the same.

The kinetic isotope effect on the first-order rate constant (measured under saturating substrate concentrations), ^D^*k*_cat_, is (nearly) temperature-independent for LOX chemistry [43]. However, for SLO-1 and h15-LOX-1, the isotope effect on the second-order rate constant, ^D^*k*_cat_/K_M_, exhibits a significant temperature dependence [89,97]. Above room temperature, the ^D^*k*_cat_/K_M_ approximates ^D^*k*_cat_ (Figure 6A). For SLO-1, the chemical step has been shown to be fully rate-determining for *k*_cat_; thus, the *k*_cat_ parameter reports on the C-H cleavage step (Figure 6B) [98]. The second-order rate constant, *k*_cat_/K_M_, reflects all steps up to and including the first irreversible step, and thus includes the initial substrate binding (E + S ⇌ ES), reorientation of substrate after the initial substrate binding, and the irreversible C-H activation (chemical) step. When ^D^*k*_cat_ and ^D^*k*_cat_/K_M_ are equivalent (as seen at elevated temperatures for LOX reactions), these parameters reflect the same transition state, ‡ (Figure 6B); in this situation, the C-H cleavage step is fully rate-determining for both first-order and second-order kinetic parameters.

Below room temperature, the value of ^D^*k*_cat_/K_M_ drops [89]. At 5 °C, the ^D^*k*_cat_/K_M_ for the LA reaction with SLO-1 is 7.7 ± 1.7, whereas the ^D^*k*_cat_ is 67 ± 5 at the same temperature (Figure 6A) [99]. The observation of ^D^*k*cat/K_M_ << ^D^*k*_cat_ under these conditions implicates an additional (partial) rate-limiting step(s) preceding the C-H cleavage step. The former has been recently attributed to a combination of substrate binding and a partially rate-limiting isomerization after substrate binds and before C-H bond cleavage [89,99]. Similar trends have been characterized for h15-LOX-1 [81,97], suggesting a potentially conserved regulatory mechanism of the rate-limiting step(s) of LOX chemistry in higher eukaryotes. 

## 4. Allosteric Regulation by Fatty Acids and Their Derivatives

### 4.1. Control of Rate-Limiting Steps

As described above, at temperatures lower than 20 °C, the ^D^*k*_cat_/K_M_ is significantly smaller than ^D^*k*_cat_ for the reaction of LA with SLO (Figure 6) or with h15-LOX-1. During preliminary inhibitor studies on SLO-1, the molecule oleyl sulfate (OS; Figure 1) was found to act as an allosteric effector [100]. Stopped-flow measurements aimed at examining the oxidation of the resting-state iron cofactor to the reactive +3-oxidation state revealed no inhibition by OS [101]. This study provided important evidence that OS binds remotely from the active site.

**Figure 7:**
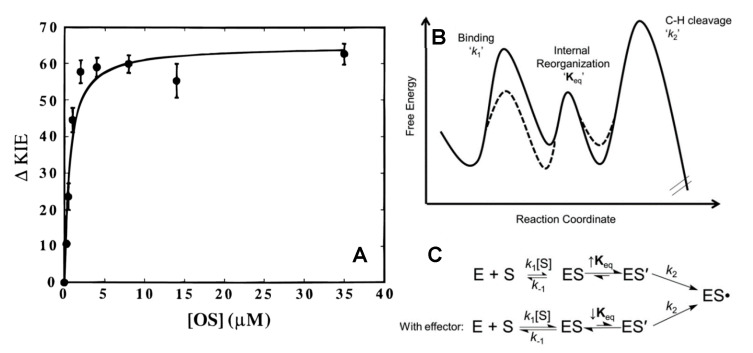
Effect of the concentration of oleyl sulfate (OS) on the change in ^D^*k*_cat_/K_M_ (∆ KIE) for SLO-1 reaction with LA at 5 °C and pH 9 (**A**) [100] (Panel A was reproduced with permission from Mogul, Johansen, and Holman. *Biochemistry* 2000). Schematic representing the impact of effector addition upon the reaction coordinate (**B**) and proposed mechanism (**C**) in soybean lipoxygenase chemistry [102] (Panels B and C are reproduced with permission from Offenbacher, Iavarone, and Klinman. *J. Biol. Chem*. 2018). In (**B**), the solid line represents the reaction coordinate (up to the first irreversible step, *k*_2_) for SLO reaction with LA in the absence of an effector. The dashed line represents the changes to the steps in the reaction coordinate in the presence of OS determined from kinetic analysis. K_eq_ refers to an internal reorganization from the initial enzyme-substrate complex ES to ES’ structure; the latter is the productive complex for chemistry.

At 5 °C, addition of OS was found to have a modest decrease (~15%) on *k*_cat_, accompanied by an 85% reduction on the second-order rate constant for protium LA [100]. Importantly, the ^D^*k*_cat_/K_M_ value was seen to rise in a hyperbolic fashion with increasing OS concentration (Figure 7A). A dissociation constant, K_d_, for OS binding to SLO-1 could be estimated from these kinetic assays as 0.6 ± 0.2 μM. The difference in the ^D^*k*_cat_/K_M_ was seen to increase by a significant amount, from ~15 to 85, under saturating OS. Under saturating OS, the magnitude of ^D^*k*_cat_/K_M_ equals the value of ^D^*k*_cat_ at 5 °C. Similarly, the ^D^*k*_cat_/K_M_ for h15-LOX-1 with LA exhibited a hyperbolic curve with OS addition (K_d_ of 0.4 ± 0.05 μM) [100].

The observation of matching isotope effects for the first- and second-order rate constants (i.e., ^D^*k*_cat_/K_M_ ≅ ^D^*k*_cat_) supports the hypothesis that substrate binding is no longer partial rate-limiting in the presence of OS at lower than room temperatures. Based on the description of the ^D^*k*_cat_/K_M_ from the microscopic rate constant analysis, the off rate of substrate binding was found to be five times faster than its on rate [100,102]. Such behavior was explained by the introduction of an additional barrier along the reaction coordinate (Figure 7B) that involves substrate reorientation within the substrate channel. From this analysis, OS addition decreases the commitment factor for catalysis (i.e., lowers the energetic barrier for substrate binding) and alters the internal equilibrium of catalytically conducive and non-productive enzyme-substrate (ES’ and ES, respectively) complexes (cf. Figure 7B,C). These effects translate to a fully rate-limiting C-H cleavage step for all temperatures in the presence of allosteric effector. Analogous to the effect of OS, strategic site-directed mutagenesis of several large aliphatic active site residues to smaller volume side chains along the putative substrate binding channel gave rise to increased off rates for substrate binding [99]. The aggregate data support that substrate binding is regulated by a two-step mechanism involving the initial binding and a substrate reorientation within the active site. At saturating substrate concentrations (or addition of allosteric effectors), the C-H cleavage step by tunneling becomes fully rate-limiting.

### 4.2. Change in Substrate Preference and Product Distribution

While AA is not a physiological substrate in plants, SLO-1 exhibits a kinetic preference for AA over LA based on the second-order rate constant ratio, (*k*_cat_/K_M_)^AA^/(*k*_cat_/K_M_)^LA^ = 1.8 [103]. Addition of the established allosteric effector, OS, to this SLO-1 reaction mixture caused an elevation in this substrate selectivity parameter, from 1.8 to 4.8 (Table 1), at fivefold its half maximal inhibitory concentration, IC_50_ [104]. As stated above, OS is exclusively an allosteric effector. Conversely, palmitoleyl sulfate (PS) is two carbons shorter than OS and results in mixed inhibition of SLO-1, with a weaker interaction for the allosteric site [101]. This effect highlights the sensitivity of the allosteric site in SLO-1. PS also increases the substrate preference ratio towards AA in SLO-1 (Table 1), though to a lesser extent and at a higher effector concentration.

For h15-LOX-2, the addition of 13*S*-HODE, the reduced enzyme product of h15-LOX-1 with LA, also gave rise to a change in substrate selectivity. Human 15-LOX-2 exhibits a pH-dependent substrate preference, in which γ-linolenic acid (GLA) is favored at physiologically relevant pH and AA is favored at slightly elevated pH values (Table 1) [105]. In the presence of 15 μM 13*S*-HODE, the h15-LOX-2 reaction with AA is activated, while the corresponding reaction with GLA is inhibited. These kinetic behaviors translate to an increased preference towards AA for both pH conditions (Table 1) [105]. Furthermore, the apparent binding strength of 13*S*-HODE was found to be responsive to both pH and the substrate, with a tighter binding for the reaction with AA. The complex kinetic data implicate a specific binding site for 13*S*-HODE and that allosteric effector binding is able to influence a substrate binding bias at the active site. For comparison, 13*S*-HODE had no significant effect on the substrate selectivity for SLO-1 [104].

**Figure 8:**
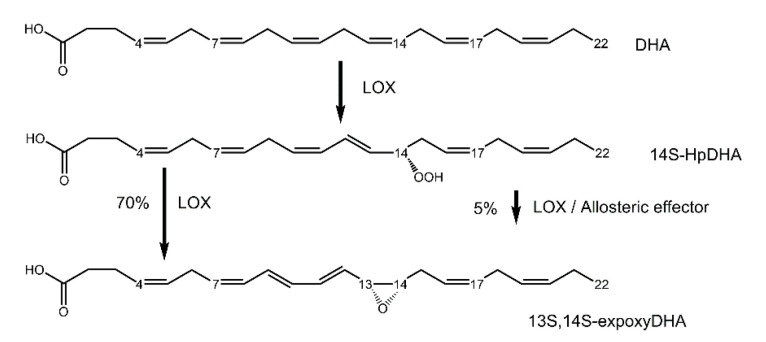
The allosteric effector, 14*S*-HpDHA, downregulates 13S,14*S*-epoxyDHA production, the maresin intermediate, from approximately 70% to below 5% at micromolar concentrations.

Human LOXs can also react with their own oxylipin products, producing multiple oxygenated molecules [7,106,107]. For example, the LOX product of DHA, 14*S*-HpDHA, can be dehydrated by both h15-LOX-1 and h12-LOX to produce 13*S*,14*S*-epoxyDHA, which is subsequently hydrolyzed to maresins [106]. Maresins are nanomolar potent oxylipins that decrease the inflammatory response, as well as promote healing and homeostasis, so their regulation has significant biological implications [108,109,110]. To this point, it was recently determined that 14*S*-HpDHA allosterically regulates the LOX production of the maresin intermediate 13S,14*S*-epoxyDHA (Figure 8). Both h15-LOX-1 and h12-LOX can either dehydrate or oxygenate 14*S*-HpDHA, with 13S,14*S*-epoxyDHA being the predominate product (approximately 70%) at low allosteric effector concentrations [106]. However, as the allosteric effector concentrations reach biologically relevant concentrations, the percentage of epoxide decreases to below 5%, outlining the possible regulation maresin production by allostery (Figure 8).

## 5. Location of the Fatty Acid Allosteric Site

### 5.1. Kinetic Properties of PLAT-deficient LOXs: Implications for the PLAT Domain

#### 5.1.1. Effect of Removal of PLAT Domain of h15-LOX-2

As shown above, the kinetic data for the addition of 13*S*-HODE to h15-LOX-2 reactions implicate a specific binding site. To begin to resolve the allosteric site without co-crystal structures available, the PLAT domain was biochemically removed, generating a stable, though slightly less active (i.e., 50% reduction in *k*_cat_), “No PLAT” h15-LOX-2 variant [105]. The removal of the PLAT domain was seen to be accompanied with a change in the substrate selectivity ratio to favor AA over GLA at pH 7.5, (*k*_cat_/K_M_)^AA^/(*k*_cat_/K_M_)^GLA^ = 1.3, even in the absence of the effector (Table 1). This result provides strong evidence that the PLAT domain does influence substrate selectivity in the native enzyme. Importantly, the binding affinity of 13*S*-HODE was seen to be nearly the same for the No PLAT and native h15-LOX-2 variants, supporting that 13*S*-HODE likely binds directly to the catalytic domain. While the PLAT domain may not be directly involved in the binding of allosteric effector, at least for 13*S*-HODE with h15-LOX-2, the aggregate data indicate an important role for the PLAT domain in the regulation of active site specificity.

#### 5.1.2. Kinetic and Structural Studies of Fungal LOXs

**Figure 9:**
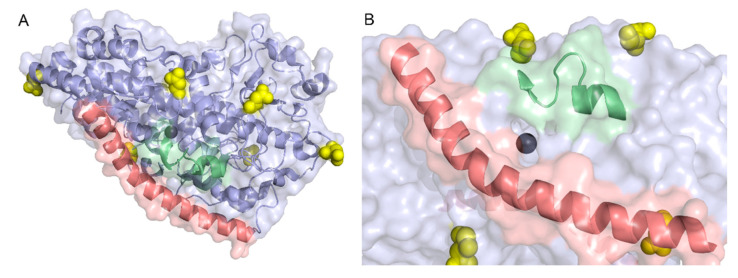
Structure of MoLOX (PDB: 5FNO [111]) is presented in (**A**). Helix 2 and the arched helix are colored as salmon and pale green, respectively. The manganese cofactor is represented as a dark gray sphere. Sites of N-linked glycosylation are shown as yellow spheres. Panel (**B**) shows the separation between helix 2 (bottom) and the arched helix (top) covering the active site. There is an expanded opening of the entrance of the substrate portal that is not seen in plant and animal LOX structures. The protein was rotated 90° relative to (**A**).

Fungal lipoxygenases are naturally deficient with an N-terminal PLAT domain (Figure 9A). These LOXs originate from pathogenic fungi that wreak havoc on many crops, including rice, wheat, and barley. The enzymes are secreted by the fungi and have been implicated in the initiation of these plant diseases [111]. In addition to lacking a PLAT domain, the fungal LOXs are quite distinct from canonical iron LOXs in several regards, including the presence of extensive N-linked glycosylation sites, use of a catalytic manganese cofactor, and an unusual bis-allylic product distribution [80,112,113].

Two fungal LOXs have emerged as model enzymes, including that from *M. oryzae* (MoLOX) and *G. graminis* (GgLOX); structures of both enzymes have been solved [111,114]. Besides the obvious lack of a PLAT domain, another divergent structural feature is an elongated helix 2 that traverses residues 79–119 (Figure 9A, salmon). This helix, even in the absence of substrate, is significantly displaced from the arched helix by ca. 13 Å, providing an open substrate portal (Figure 9B). These structural properties provide a framework for MoLOX’s ability to accommodate large, bulky phospholipid substrates and distinctive kinetic properties [83,115].

Extensive kinetic analysis determined that first- and second-order rate constants (and their isotope effects) for the MoLOX reaction with LA are completely independent of pH [83]. At pH 9, the values of ^D^*k*_cat_/K_M_ for the reaction of dideuterated (11,11-D_2_) LA were equivalent to ^D^*k*_cat_ values (60–70) within the accessible temperature range explored (10–30 °C). This kinetic behavior is exclusive to fungal LOXs and there is no evidence for allosteric regulation of MoLOX by fatty acids. 

### 5.2. Identification of pKa in Effector Binding: Putative Role for Histidine

Using a competitive HPLC assay [97], the substrate selectivity ratio, (*k*_cat_/K_M_)^AA/^(*k*_cat_/K_M_)^LA^, for h15-LOX-2 was found to be sensitive to pH, increasing from 1.4 ± 0.3 at pH 6.0 to 4.5 ± 0.5, at pH 10 [81]. These data were fit to a sigmoidal curve yielding a pK_a_ of 7.7 ± 0.1. Addition of 13*S*-HODE (Figure 1), an allosteric effector for h15-LOX-2, did not alter the substrate preference at pH 7.5. However, at pH 8.5 and 10, the substrate selectivity ratio dropped to 1.8 ± 0.3 and 1.9 ± 0.3, respectively. This behavior was rationalized by a saturated allosteric site at pH 7.5 associated with tight binding (K_d_ ≈ 5 nM) [81]. At pH 8.5 and above, the binding was estimated to be at least tenfold weaker. The extrapolated binding constants were interpreted as a solvent exposed histidine that hydrogen bonds with the carboxylate head group of 13*S*-HODE upon protonation of the histidine. 

### 5.3. Docking Model

**Figure 10:**
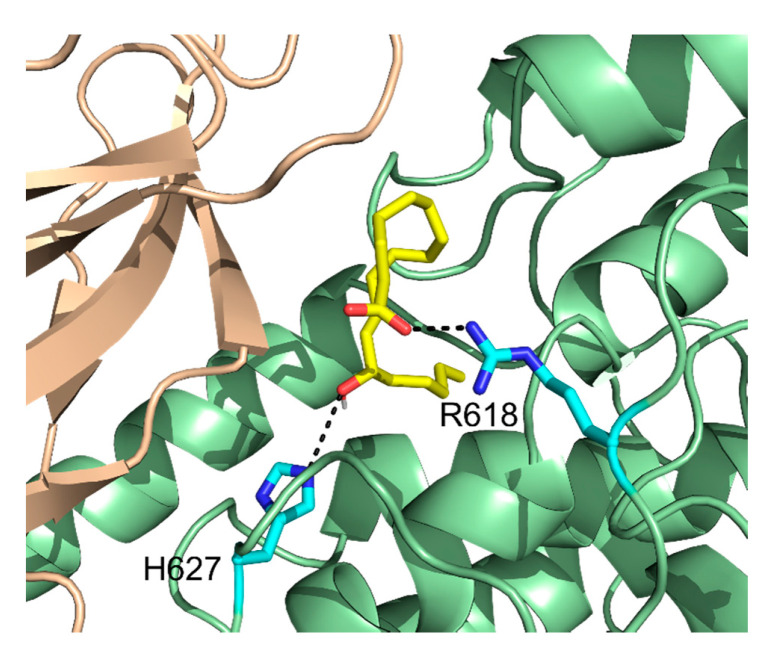
In silico model of human 15-LOX-2 structure (PDB: 4NRE) docked with 13*S*-HODE (yellow sticks) in the proposed allosteric site at the intersection between the PLAT (wheat) and catalytic (light green) domains [81]. The structure has been rotated 180° from Figure 2B. Potential hydrogen bond interactions are depicted by dashed lines and side chains labeled.

While crystal structures have been solved for several LOXs, including those presented in Figure 2, the co-crystallization of LOXs with an allosteric effector has remained elusive. In an effort to gain structural insight into the location of the allosteric site, molecular docking has been employed for h15-LOX-2 [81]. The information gleaned from the pH-dependent study was previously exploited to narrow down the potential binding sites for 13*S*-HODE. Of the eight solvent exposed histidine residues in h15-LOX-2, only three, positioned in the catalytic domain, lined the site in which 13*S*-HODE could be docked: H376, H394, and H627 (Figure 10). From the docking results, the most probable interaction places the carboxylate group of 13*S*-HODE in hydrogen bonding with histidine 627. The hydroxyl group along the fatty acyl chain forms hydrogen bonding interactions with arginine 407 and tyrosine 408 of the catalytic domain in this model. This putative allosteric site lies in a crevice at the intersection between the PLAT and allosteric domains. However, no such docking model has been reported for allosteric effectors with SLO-1.

This model is further supported by a recent structure of human 5-LOX bound to an isoform-selective allosteric effector, AKBA (3-acetyl-11-keto-beta-boswellic acid), that alters the substrate regio-specificity of the enzyme [38]. The AKBA molecule was seen to associate at the interface of the PLAT and catalytic domains in h5-LOX.

## 6. Long-Range Allosteric Network

### 6.1. Multi-Temperature HDX-MS

Hydrogen-deuterium exchange coupled with mass spectrometry (HDX-MS) was employed to study the allosteric network in SLO-1 stemming from the interaction with OS [102]. HDX-MS reports on the exchange of hydrogen in amide N-H bonds with deuterium. The technique has served as a powerful tool in defining protein networks relevant to allostery [116,117,118,119,120]. Under EX-2 conditions, in which chemical exchange (i.e., N-H → N-D) is slower than the closing rate of the transient amide exposure to solvent, HDX-MS provides a thermodynamic read-out of local, transient fluctuations of the peptide backbone [121,122]. Samples are prepared by diluting the protein (1:10 by volume) into deuterated buffer and the samples are placed into a temperature-controlled water bath. At a specific time, a sample is removed from the bath and rapidly cooled and acid is added (pH 2.4) to “quench” the exchange [121,122]. Samples are then digested with pepsin and the resulting peptides are analyzed by LC-MS. 

**Figure 11:**
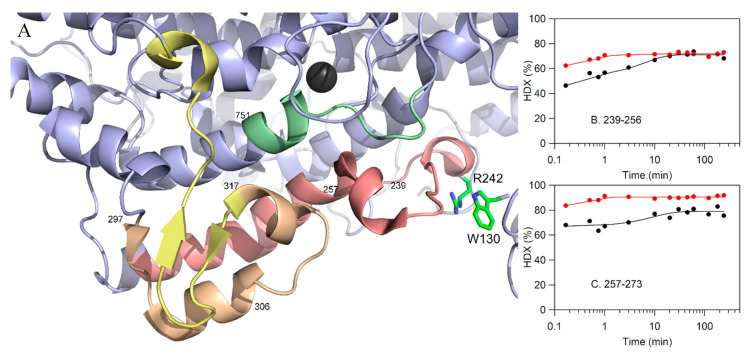
Partial structural model of the SLO-1 catalytic domain (**A**) with peptides affected by the presence of OS, color-coded as follows: residues 239–256 and 257–273 (salmon), 297–305 and 306–316 (wheat), 317–334 (pale yellow), and 751–761 (pale green). The number of the N-terminal amino acid of each peptide, affected by OS, is shown in the structure for reference. The orientation of SLO-1 has been rotated by 180° relative to the structure in Figure 2A. A cation-π interaction, involving R242 (catalytic domain) and W130 (PLAT domain), plays an important role in communicating allostery (see Section 6.2). (**B** and **C**) HDX-MS traces, collected at 10 °C, for peptides encompassing helix 2 (colored salmon in (**A**)). The color coding of the HDX-MS traces represents SLO in the absence of OS (black) and in the presence of OS (red). Data are corrected for deuterium content and peptide-specific back-exchange [102,123].

Peptic digests of SLO-1 resulted in approximately 300 reproducible peptides, providing 94% coverage of the entire primary sequence [27]. EX-2 conditions were confirmed for these peptides from the characteristic unimodal, time-dependent shifts of the isotopic distribution in the mass spectra [123]. HDX samples were prepared in the absence and presence of 10 μM OS, in which the allosteric site is expected to be saturated (K_d_ = 0.6 ± 0.3 μM) [100]. Examination of 46 non-overlapping peptides revealed the most striking differences in the catalytic domain for data collected at 10 °C [102]. The behavior manifests as enhanced apparent rates of hydrogen exchange (Figure 11B,C). The greatest impact was upon helix 2 (residues 257–273); at 10 °C, the addition of OS causes a 25-fold increase in the rate of exchange and a greater extent of deuterium exchange at equilibrium (at 4 h). The former reflects enhanced peptide fluctuations that promote a more rapid apparent exchange, while the latter is consistent with an induced conformational change that exposes additional amide hydrogen(s) for exchange. Importantly, peptide 257–273 contains the π-helix of SLO-1 that is anticipated to gate substrate binding.

The HDX-MS results provide a framework for the mechanistic implications of OS inferred from kinetic analysis (cf. Section 4.1 and Figure 7). The emerging view is that OS presumably binds at the surface of the catalytic domain at the interface of the PLAT domain. The binding of the allosteric molecule causes enhanced fluctuations (and a conformational change) of helix 2 that lines the substrate entrance portal. The enhanced flexibility of helix 2 (Figure 11) is linked to increased on and off microscopic rate constants for substrate binding and thereby, OS alleviates the partial rate-limiting substrate binding at below room temperatures (Figure 7). 

HDX experiments were also conducted at 20, 25, 30, and 40 °C [102]. At room temperature and above, the apparent rates of deuterium exchange for helix 2 peptides with OS were too fast within the dynamic range of the HDX-MS experiments to detect differences between the presence and absence of OS. The lower temperature (10 °C) enabled quantitative assessment of the impact of OS on the dynamic nature of SLO-1. This study underscores the advantages of the multi-temperature HDX (mtHDX) approach [27] for the detection of protein allostery.

### 6.2. Role of a Cation-π at the Domain Intersection

**Figure 12:**
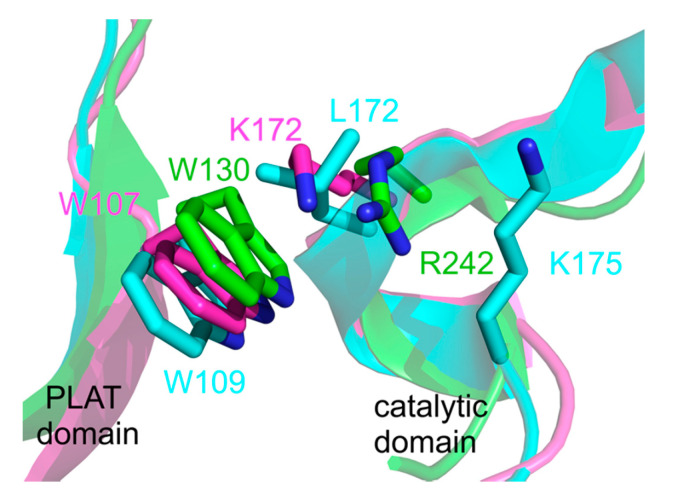
Diagram of the putative cation-π interaction that connects the PLAT domain to helix 2 of the catalytic and that has been implicated [54] in Ca^2+^-dependent activation in 11*R*-LOX. The structural overlays represent the coral 11*R*-LOX (purple; PDB: 3O8Y), h15-LOX-2 (cyan; PDB: 4NRE), and SLO-1 (green; PDB: 3PZW) crystal structures.

If the allosteric regulation of LOX is influenced by the PLAT domain, how are the dynamics communicated to the catalytic domain? Coral 11R-LOX is one of the most highly regulated lipoxygenases. Activity is completely contingent upon Ca^2+^ binding at the PLAT domain and membrane association [124]. Further, the 11*R*-LOX crystal structure, similar to that of h5-LOX [37], shows an occluded active site, in which Ca^2+^-dependent membrane binding (at the PLAT domain) is anticipated to trigger a conformational change in helix 2 (in the catalytic domain) to permit substrate access [54]. From the 11*R*-LOX structure (PDB: 3O8Y), a conserved Trp was identified in the PLAT domain that lies at the interface of the catalytic domain (Figure 12). For both the coral 11*R*-LOX and h5-LOX, there is a cationic residue (K172 in 11*R*-LOX and R165 in 5-LOX) spatially adjacent to the tryptophan in the PLAT domain and positioned at the end of helix 2 in the catalytic domain. It has been proposed that this cation-π interaction may be essential to the long-range allosteric control of 11*R*- and h5-LOX reactivity by calcium [54,125]. Non-conservative mutations of the tryptophan or cationic residue resulted in decreased catalytic proficiency (by about tenfold), with little-to-modest impact on the structural stability of the enzyme variants [126]. How these LOX variants influence allosteric activation, however, is not yet fully understood.

Sequence alignment of several plant and animal LOXs indicated complete conservation of a tryptophan at the homologous position of W107 in 11*R*-LOX [54]. The homologous residues are W130 in SLO-1 and W109 in h15-LOX-2 (Figure 12). While this structural feature is conserved in plants, SLO-1 (and other plant LOX) reactivity is not greatly influenced by Ca^2+^ [57], yet an arginine, R242, in SLO-1 is seen to form a cation-π interaction with W130, bridging the PLAT and catalytic domains (Figure 11A and Figure 12). From the HDX-MS study of SLO-1 described above [102], this cation-π interaction has been proposed to mediate the effector-induced, enhanced flexibility of helix 2 (Figure 11) that is responsible for regulating the rate-limiting steps of catalysis [100]. The physiological relevance of this allosteric regulation in soybean growth or development has not yet been established.

Contrarily in the crystal structure of h15-LOX-2, the cationic residue, K175, is in a “flipped out” conformation and an aliphatic residue, L172, is positioned within van der Waals distance to W109 (Figure 12). This h15-LOX-2 structure is among the few solved with a substrate mimic in its active site. Substrate binding could cause a shift in the conformation of helix 2. An HDX-MS study of h15-LOX-2 demonstrated that helix 2 is highly exchanged (≥90%); the exchange behavior was also seen to be responsive to addition of substrate at the active site [56]. While the peptide appears well-structured from X-ray crystallography (Figure 2), the HDX properties of helix 2 is consistent with a highly flexible propensity. Similar HDX properties were reported for the helix 2 in SLO-1 [102]. While the tryptophan may be highly conserved for plant and animal LOXs, the structural difference for h15-LOX-2 may unlock the possibility that the (re)positioning or flexibility of the cationic residue may serve to trigger the allosteric control of helix 2 conformation and its dynamics. Like SLO-1, Ca^2+^ and membrane association does not appear to be a significant determinant for h15-LOX-2 reactivity. Therefore, this conserved cation-π interaction could serve as a general allosteric control “switch” to regulate LOX catalysis. The access to more crystal structures coupled with functional analysis of strategic site-directed mutants using comparative kinetic isotope effects on the first- and second-order rate constants could help to resolve these open queries.

## 7. Conclusions

In higher eukaryotes, the initial and often rate-limiting C-H activation step of fatty acid oxidation by LOXs is allosterically regulated by fatty acids and their derivatives. These fatty acid derivatives may also influence substrate selectivity and/or product distribution. The accumulating data from kinetic analysis of substrate selectivity and comparative isotope effects, X-ray crystallography and hydrogen-deuterium exchange of the model plant (SLO-1) and human (h15-LOX-1/2) enzymes support the hypothesis that the allosteric effector binding originates at or near the N-terminal, regulatory PLAT domain. This allosteric interaction elicits altered conformations and/or enhanced structural plasticity of helix 2 in the catalytic domain via a conserved cation-π structural feature, with helix 2 expected to serve as a gatekeeper for substrate binding. As summarized herein, this combinatorial application of biophysical tools is an excellent approach to resolve the allosteric regulation of lipoxygenase C-H activation chemistry and could provide guidelines for the development of effective allosteric molecules to be leveraged as LOX isoform-selective drugs, which can tune substrate selectivity and/or product distribution of LOX to quell the inflammatory process.

## Figures and Tables

**Table 1:** Substrate selectivity for SLO-1 and h15-LOX-2 with and without the allosteric effector.

	(*k*_cat_/K_M_)^AA^/(*k*_cat_/K_M_)^LA^	IC_50_ (μM)
SLO-1	1.8 ± 0.2	N.A. ^a^
+OS	4.8 ± 0.3	1.0 ± 0.1
+PS	4.0 ± 0.3	13 ± 2
	(*k*_cat_/K_M_)^AA^/(*k*_cat_/K_M_)^GLA^	
	pH 7.5	pH 8.5
h15-LOX-2	0.63 ± 0.04	2.1 ± 0.2
+13*S*-HODE	2.3 ± 0.3	6.7 ± 0.8
No PLAT	1.3 ± 0.2	3.6 ± 0.3

^a^ N.A.: nothing added.
